# Using deep LSD to build operators in GANs latent space with meaning in real space

**DOI:** 10.1371/journal.pone.0287736

**Published:** 2023-06-29

**Authors:** J. Quetzalcóatl Toledo-Marín, James A. Glazier

**Affiliations:** 1 Luddy School of Informatics, Computing and Engineering, Biocomplexity Institute, Indiana University, Bloomington, IN, United States of America; 2 TRIUMF, Vancouver, BC, Canada; Shanghai Maritime University, CHINA

## Abstract

Generative models rely on the idea that data can be represented in terms of latent variables which are uncorrelated by definition. Lack of correlation among the latent variable support is important because it suggests that the latent-space manifold is simpler to understand and manipulate than the real-space representation. Many types of generative model are used in deep learning, *e.g*., variational autoencoders (VAEs) and generative adversarial networks (GANs). Based on the idea that the latent space behaves like a vector space Radford et al. (2015), we ask whether we can expand the latent space representation of our data elements in terms of an orthonormal basis set. Here we propose a method to build a set of linearly independent vectors in the latent space of a trained GAN, which we call quasi-eigenvectors. These quasi-eigenvectors have two key properties: i) They span the latent space, ii) A set of these quasi-eigenvectors map to each of the labeled features one-to-one. We show that in the case of the MNIST image data set, while the number of dimensions in latent space is large by design, 98% of the data in real space map to a sub-domain of latent space of dimensionality equal to the number of labels. We then show how the quasi-eigenvectors can be used for Latent Spectral Decomposition (LSD). We apply LSD to denoise MNIST images. Finally, using the quasi-eigenvectors, we construct rotation matrices in latent space which map to feature transformations in real space. Overall, from quasi-eigenvectors we gain insight regarding the latent space topology.

## Introduction

*Generative models* (GMs) are a class of Machine Learning (ML) model which excel in a wide variety of tasks [1]. The optimization of a GM finds a function G that maps a set of *M* *latent variables* in *latent space* to a set of *d* variables in real space representing the data of interest (*e.g*., sets of images, music, videos, *etc*.), *i.e*. $G:R^{M}\to R^{d}$ where *d* >> *M* > 1. When building a GM, we first define the support of the latent variables, then obtain the function G by iteratively optimizing a loss function. Loss function choice depends on application, *e.g*., maximum log-likelihood is common in Bayesian statistics [2], Kullback–Leibler divergence is common for variational autoencoders (VAEs) [3, 4], and the Jensen-Shannon entropy and the Wasserstein distance are common with generative adversarial networks (GANs) [5–9]. The last two models are deep learning models. Deep learning is a field in artificial intelligence which has had great success in recent years and has pervaded many fields in science and health as well as our day to day lives. When we fit a latent variable model to a data set, we are finding a description of the data in terms of “independent components”. Latent variables, |*z*_*i*_〉, have a simple distribution, often a separable distribution (*i.e*., $P\left({\left\{z_{i}\right\}}_{i=1}^{M}\right)=\prod_{i=1}^{M}P\left(z_{i}\right)$) [2]. Often the latent representation of data lives in a simpler manifold than the original data while preserving *relevant* information. There are many examples of latent representation used to understand or describe more complicated features, ranging from statistical methods like Latent Class Analysis to examples in statistical physics and condensed matter such as order parameters for phase classification and even the long standing problem of the genotype-phenotype where the genome is taken as the latent representation of the phenotype. Other examples are, for instance, Ref. [10] proposes a time-frequency representation of a signal that allows the reconstruction of the original signal, which relies in what they define as “*consensus*”. Their proposed method generates sharp representations for complex signals.

Deep neural networks can function as surrogate propagators for time evolution of physical systems [1]. While the latent variables are constructed to be independent identically distributed (i.i.d.) random variables, the training process *entangle* these latent variables. Latent variable disentanglement is an active area of research employing a wide variety of methods. For instance, in Ref. [11], the authors train a GAN including the generator’s Hessian as a regularizer in the loss function, leading, in optimum conditions, to linearly independent latent variables, where each latent variable independently controls the strength of a single feature. Ref. [12] constructs a set of *quantized* vectors in the latent space using a VAE, known as *vector quantized variational autoencoder* (VQ-VAE). Each *quantized* vector highlights a specific feature of the data set. This approach has been used in OpenAI’s jukebox [13]. A major drawback of these approaches is the lack of freedom in relating specific features in real space with specific latent space directions. This can be overcome by *conditionalizing* the generative model [14]. However, conditionalization can reduce the latent space *smoothness* and *interpolation capacity*, since the condition is usually enforced by means of discrete vectors as opposed to a continuous random latent vector. Diffusion-based models [15] have shown they can equate to GANs in performance and have become highly popular in recent times.

**Fig 1:**
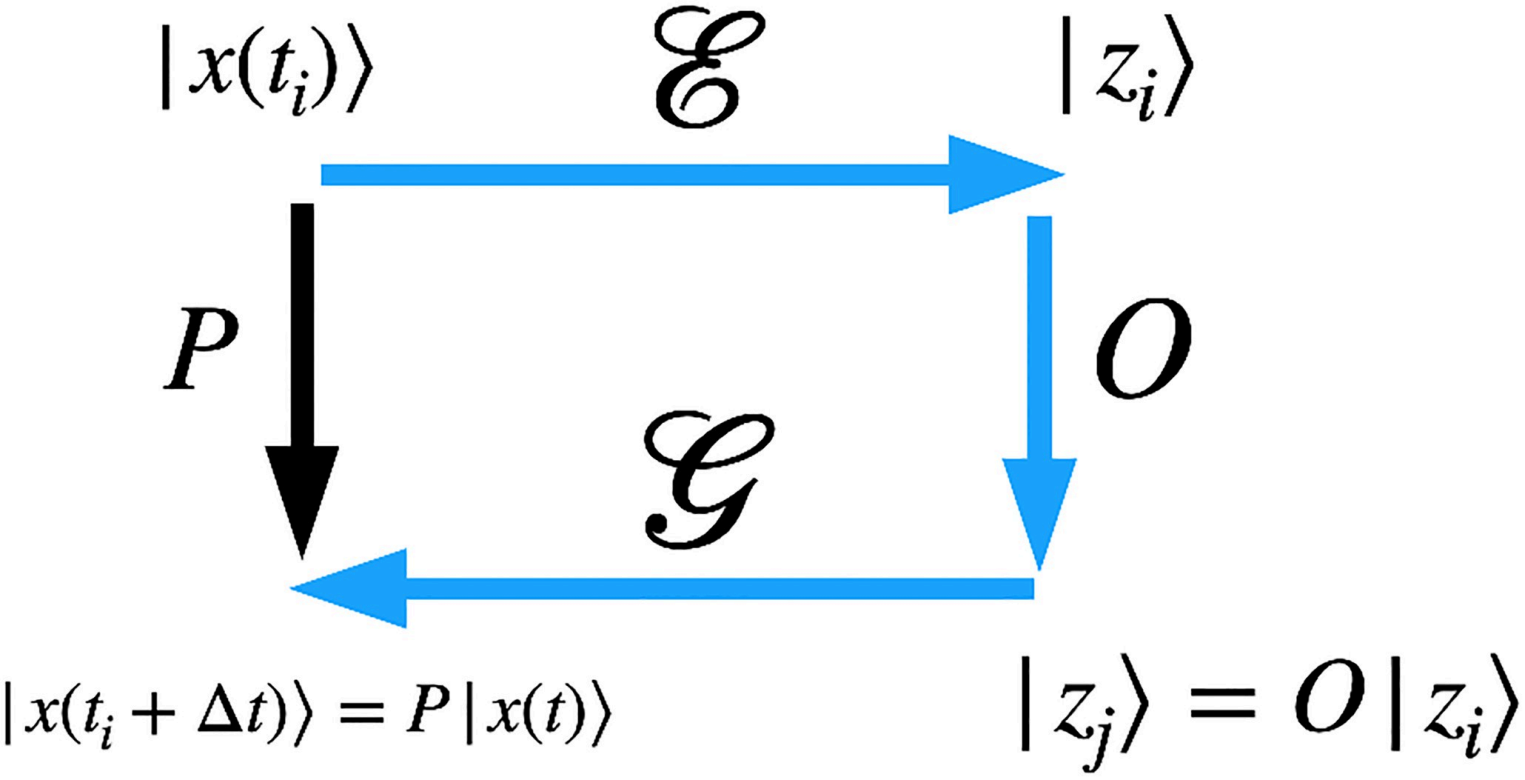
Schematic of spaces and operators. *P* is an operator in real space that evolves the state |*x*(*t*_*i*_)〉 to |*x*(*t*_*i*_ + Δ*t*)〉. E is an Encoder and G is the Generator that maps latent variables to real space. *O* is an operator in latent space. The black arrow shows the time propagation done by applying the operator *P* to |*x*(*t*_*i*_)〉 which yields |*x*(*t*_*i*_ + Δ*t*)〉. The blue arrows show the path where the data |*x*(*t*_*i*_)〉 gets encoded into latent space, |*z*_*i*_〉, then the operator *O* is applied to the latent vector yielding a new latent vector |*z*_*j*_〉. Finally, the new latent vector get decoded and yields |*x*(*t*_*i*_ + Δ*t*)〉.

Here we propose a method to relate a specific chosen labeled feature with specific directions in latent space such that these directions are linearly independent. Having a set of linearly-independent latent vectors associated with specific labeled features allows us to define operators that act on latent space (*e.g*. a rotation matrix) and correspond to feature transformations in real space. For instance, suppose a given data set in real space corresponds to the states of a molecular dynamic simulation, *i.e*., the *i*-th data point in the data set can be the positions of the molecules at time *t*_*i*_, |*x*_*i*_〉 → |*x*(*t*_*i*_)〉, where |*x*(*t*_*i*_)〉 is a vector. Let us suppose that $|x\left(t_{i}\right)\rangle =G|z_{i}\rangle$ and $|x\left(t_{i}+\Delta t\right)\rangle =G|z_{j}\rangle$, as depicted in Fig 1. How can we construct an operator in latent space, $O_{\Delta t}$, such that $|z_{j}\rangle =O_{\Delta t}|z_{k}\rangle$?. For this construction to be possible, we argue the operator G must be *locally* linear. Furthermore, in order to build the operator O, we need a basis that **spans** latent space. While linearity might seem counterintuitive given how NNs work, growing evidence suggests such linearity in practice. For instance, on the one hand there is an ongoing debate on how deep should a NN be to perform a specific task, on the other hand, it has been proposed the equivalence between deep NNs and shallow wide NNs [16]. For at least one image-related GAN, simple vector arithmetic in latent space leads to feature transformations in real space (*e.g*., removal of sunglasses, change in hair color, gender, *etc*.) [17]. However, a complete understanding on how specific features in real space map to latent space and how are these features arranged in latent space (*latent space topology*) or why some GANs’ latent space behave like linear operators is lacking. It is believed that the latent representation of data with a given labeled feature forms a cluster. However, the tools employed to show this clustering effect quite often consist in a dimensional reduction *e.g*., t-SNE [18] which collapses the latent representation into two or three dimensions. Other methods include principal component analysis, latent component analysis and important component analysis [2, 19, 20]. Our method does not collapse or reduce the latent space, allowing us to inspect latent space topology by spanning all latent space directions. We strongly believe the need of a set of basis vectors for understanding the topology of the latent space. Given the typical high-dimensionality of the latent space, we employ the Gram-Schmidt method to construct linearly independent vectors from a set of vectors that map to specific features. This approach enables us to visualize the feature entanglement in the latent space. We contend that our work contributes to a better understanding of latent space topology in two key ways: 1) through the method itself, which involves constructing a set of basis vectors in the latent space that map to specific features in the real space using Gram-Schmidt, and 2) by possessing the latent space basis vectors that map to specific features in the real space, which enables data manipulation in the latent space via linear algebra. As a proof of concept, we demonstrate the method by applying it to MNIST.

In the next section we introduce our mathematical method and notation and apply the method to the MNIST data set. In the Results section we show how we can use this method to understand the topology of the latent space by performing classification via principal component analysis; we apply this method to denoise images; and finally we show how to perform matrix operations in latent space which map to image transformations in real space. We discuss future steps and limitations in the last section.

## Methods and materials

Assume a vector space which we call real space and denote the vectors in this space |*x*〉 with |*x*〉 ∈ ℜ^*d*^. Assume a set $\{|x_{i}\rangle {\}}_{i=1}^{N}$, which we call the dataset with *N* the dataset size. Similarly, we assume a vector space, which we call the latent space and denote these vectors |*z*〉 with |*z*〉 ∈ ℜ^*M*^ (in general, *M* ≤ *d*). We also consider three deep neural networks, a Generator G, an Encoder E and a Classifier C. We can interpret G as a projector from latent space to real space, *i.e*., $|x_{i}\rangle =G|z_{i}\rangle$, and interpret E as the inverse of G. However, this last statement has to be taken with a grain of salt, due to how variational autoencoders work. In fact, if $|z_{a}\rangle =E|x_{i}\rangle$ and $|z_{a^{\prime }}\rangle =E|x_{i}\rangle$, in general, |*z*_*a*_〉 ≠ |*z*_*a*′_〉, since these vectors are i.i.d. random vectors, sampled from a Gaussian distribution with mean and standard deviation dependent on |*x*_*i*_〉 (the correct mathematical notation to use would be $|z_{a}\rangle ∼N(|\mu \rangle ,|\sigma \rangle )$, where N is a multivariate Gaussian distribution with mean and standard deviation |*μ*〉 and |*σ*〉, respectively, which depend on $E|x_{i}\rangle$) [4]. Finally, the Classifier projects real-space vectors into the label space, *i.e*., $|y_{k}\rangle =C|x_{i}\rangle$, where |*y*_*k*_〉 ∈ *L*, where *L* denotes the label space. We assume that each vector |*y*_*k*_〉 is a one-hot-vector. The length of |*y*_*k*_〉 equals the number of labels |*L*| = *l* and *k* = 1, …, *l*. Henceforth, we assume that *l* < *M*.

We define $\{|\xi_{i}\rangle {\}}_{i=1}^{M}$ to be a set of basis vectors in latent space such that the inner product between them yields 〈*ξ*_*i*_|*ξ*_*j*_〉 = *Cδ*_*ij*_, where *C* is the norm and *δ*_*ij*_ is the Kronecker delta function. Henceforth we call the set of basis vectors $\{|\xi_{i}\rangle {\}}_{i=1}^{M}$ the *quasi-eigenvectors* since they form a basis and each one represents a feature *state* in latent space. Notice that we can define the operator $A=\sum_{j=1}^{M}|\xi_{j}\rangle \langle \xi_{j}|$ (here |*κ*〉〈*γ*| denotes the outer product between vectors |*κ*〉 and |*γ*〉), which implies $A|\xi_{i}\rangle =C|\xi_{i}\rangle$. Any vector in latent space can be expressed as a linear superposition of these quasi-eigenvectors, *viz*,

$$\begin{array}{c} \left|z\right\rangle =\sum_{j=1}^{M}c_{j}\left|\xi_{j}\right\rangle \;. \end{array}$$ (1)

where |*c*_*i*_| = |〈*ξ*_*i*_|*z*〉| is the amplitude of |*z*〉 with respect to |*ξ*_*i*_〉 and gives a measure of |*z*〉’s projection with the quasi-eigenvector |*ξ*_*i*_〉. Constructing a set of basis vectors is straightforward. However, we wish each labeled feature to corresponds one-to-one with a quasi-eigenvector. Since we are assuming that *l* < *M*, there will be a set of quasi-eigenvectors that do not correspond to any labeled feature.

To obtain a set of orthogonal quasi-eigenvectors, we use the Gram-Schmidt method. Specifically:

1. We train the GAN, which is composed by two NNs, namely, the Generator and the Discriminator, using the training set $\{|x_{i}\rangle {\}}_{i=1}^{N}$ as in Ref. [6].
2. We train the Classifier independently, using the training set.
3. We train a VAE using the trained Generator as the decoder. We also use the Classifier to classify the output of the VAE. We include in the loss function a regularizer $\lambda \cdot L_{class}$, where λ is a hyperparameter and $L_{class}$ denotes the Classifier’s loss function. At this stage, we only train the Encoder, keeping the Generator and Classifier fixed. There are several options to choose from for the $L_{class}$ loss function. In our case, we used the Cross Entropy with a softmax activation function, *i.e*.,
  $$\begin{array}{c} L_{class}\left(|y\right\rangle ,|y^{GT}\rangle )=-\sum_{i=1}^{L}y_{i}^{GT}\;\text{log}\;\frac{e^{y_{i}}}{\sum_{j=1}^{L}e^{y_{j}}}\;, \end{array}$$ (2)
  where *y*_*i*_ and $y_{i}^{GT}$ are the *i*th components of the vectors |*y*〉 and |*y*^*GT*^〉, respectively, and |*y*^*GT*^〉 is the ground truth vector.
4. Define *n* to be an integer such that *M* = *n* × *l*. Then, for each label, we allocate *n* sets of latent vectors and we denote each of these latent vectors as $|z_{\alpha ,i}^{k}\rangle$, where *α* denotes the label, *i* = 1, …, *n* and *k* = 1, …, *V*. Here *V* is the number of elements (latent vectors) in each set corresponding to the pair (*i*, *α*) ∈ *n* × *l*. We build each of these sets $\{|z_{\alpha ,i}^{k}\rangle {\}}_{k=1}^{V}$ in two ways:
  - Using the training set, we encode each vector $|x_{a}\rangle \to |z_{a}\rangle =E|x_{a}\rangle$, then we decode the latent vector, *i.e*., $|z_{a}\rangle \to |x_{a}\rangle =G|z_{a}\rangle$, and then we classify the output, *i.e*., $|x_{a}\rangle \to |y_{a}\rangle =C|x_{a}\rangle$. For each label *l*, there is a set of latent vectors. The goal is to have a large number of the latent vectors representation of the data set arranged by label. Due to the large latent space dimensionality, we may require additional latent vectors besides those generated directly by encoding the training set. For this reason, we do the following.
  - We generate random latent vectors and map each of these latent vectors to their labels using the Generator and the Classifier as in 4(a), *i.e*., once we generated the random latent vector |*z*_*a*′_〉 using a random multivariate Gaussian generator, we project it to real space $|z_{a^{\prime }}\rangle \to |x_{a^{\prime }}\rangle =G|z_{a^{\prime }}\rangle$, and then we classify the output, *i.e*., $|x_{a^{\prime }}\rangle \to |y_{a^{\prime }}\rangle =C|x_{a^{\prime }}\rangle$. Notice that with this approach we can generate as many latent vectors as desired.
  We denote as *V* the number of latent vectors per set (*i*, *α*).
5. We take the average over *V* for each set of latent vectors $\{|z_{\alpha ,i}\rangle {\}}_{k=1}^{V}$ and denote that average |*η*〉_*α*,*i*_, *i.e*.,
  $$\begin{array}{c} {\left|\eta \right\rangle }_{\alpha ,i}=\frac{1}{V}\sum_{j=1}^{V}\left|z_{\alpha ,i}^{j}\right\rangle \;. \end{array}$$ (3)
  It is worth noticing that since the latent vectors are sampled from a multivariate Gaussian distribution, the average |*η*_*α*,*i*_〉 is finite and unbiased. By defining operators in latent space in terms of outer products of the |*η*_*α*,*i*_〉 vectors, these latent space operators will have encoded in them the set of latent vectors $|z_{\alpha ,i}^{k}\rangle$.
6. To impose orthogonality, we use the Gram-Schmidt method. Thus, from the vectors |*η*_*α*,*i*_〉 we generate a set of quasi-eigenvectors |*ξ*〉_*α*,*i*_, *i.e*.,
  $$\begin{array}{ccc} {\left|\xi \right\rangle }_{1,1} & = & {\left|\eta \right\rangle }_{1,1} \end{array}$$ (4)
  $$\begin{array}{ccc} {\left|\xi \right\rangle }_{2,1} & = & {\left|\eta \right\rangle }_{2,1}-\frac{{}_{2,1}{\left\langle \eta |\xi \right\rangle }_{1,1}}{{}_{1,1}{\left\langle \xi |\xi \right\rangle }_{1,1}}{\left|\xi \right\rangle }_{1,1} \end{array}$$ (5)
  $$\begin{array}{c} \ldots \end{array}$$ (6)
  $$\begin{array}{ccc} {\left|\xi \right\rangle }_{l,n} & = & {\left|\eta \right\rangle }_{l,n}-\sum_{\alpha =1}^{l-1}\sum_{i=1}^{n-1}\frac{{}_{l,n}{\left\langle \eta |\xi \right\rangle }_{\alpha ,i}}{{}_{\alpha ,i}{\left\langle \xi |\xi \right\rangle }_{\alpha ,i}}{\left|\xi \right\rangle }_{\alpha ,i}\;. \end{array}$$ (7)
  Such that:
  $$\begin{array}{c} {}_{\alpha ,i}{\left\langle \xi |\xi \right\rangle }_{\beta ,j}=C\delta_{\alpha \beta }\delta_{ij} \end{array}$$ (8)

In Eq (8), *C* is the value of the norm. The set of quasi-eigenvectors ${\{|\xi \rangle }_{\alpha ,i}{\}}_{\alpha =1,i=1}^{l,n}$ span the latent space and, as we will show, a subset of them map to specific features.

The key point is that the set of quasi-eigenvectors form a basis set in latent space and each direction corresponds to a feature in real space. This structure allows us to give a better topological description of latent space, *i.e*., how does labeled features map to latent space similar to how molecular configurations map to the *energy landscape* [21]. In addition, we can use the set of quasi-eigenvectors as tools for classification, denoising and topological transformations. We demonstrate these applications next using the MNIST dataset.

### Applying method to MNIST

**Fig 2:**
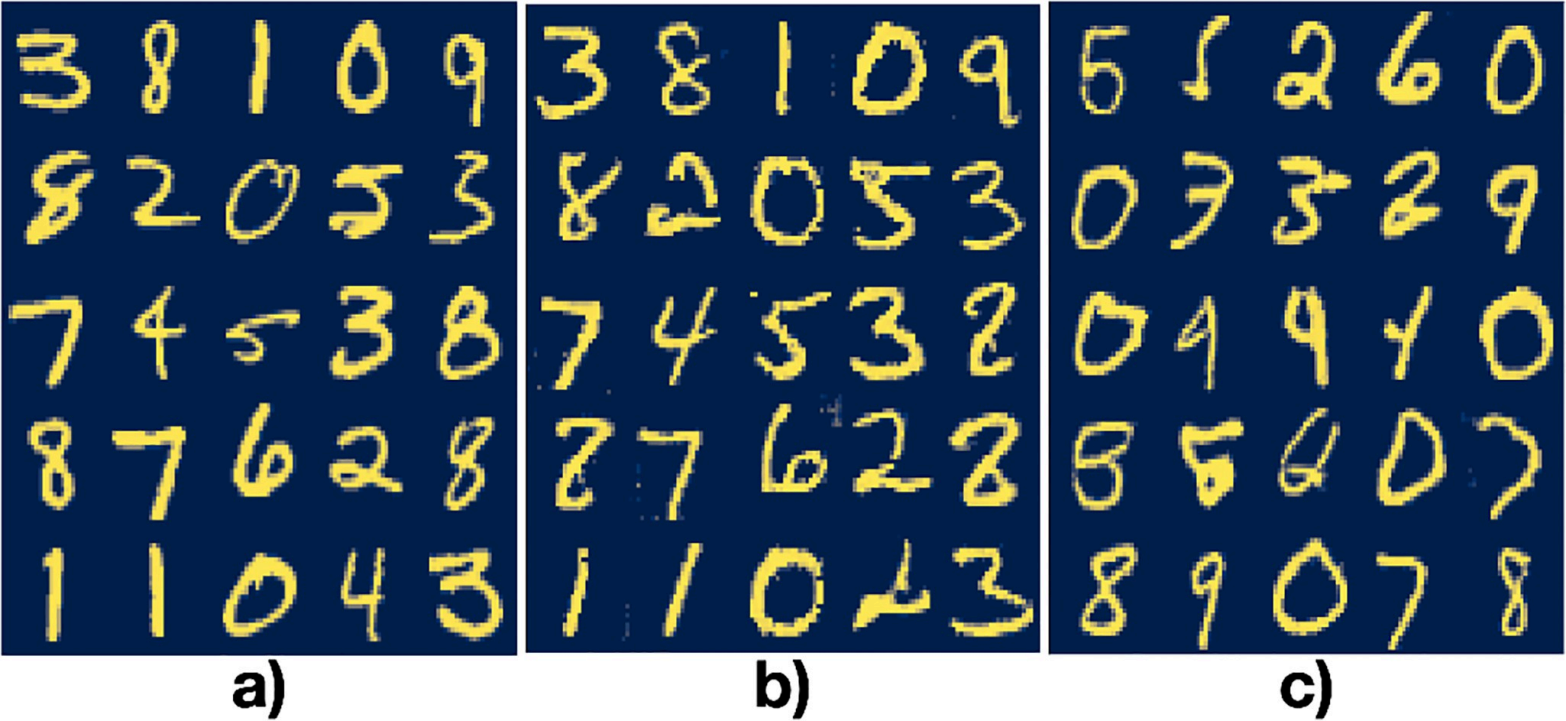
A batch from the a) dataset, b) the same dataset encoded and decoded using the Generator as Decoder and c) random latent vectors given as input to the Generator.

We trained a GAN, a Classifier and a VAE using the MNIST dataset which has 60*k* and 10*k* one-channel images in the training and test set, respectively, with dimensions 28 × 28 pixels. In Fig 2a we show a sample of the dataset. The MNIST dataset can be found in many machine learning packages (*e.g*., PyTorch, Flux for Julia, etc.) as well as in [22]. We fixed the batch size to 25 and number of epochs to 500 during all training runs. We trained the GAN using the training set, used the Jensen-Shannon entropy as the loss function [6], the ADAM optimizer with hyperparameters *η* = 0.0002, *β*_1_ = 0.9, *β*_2_ = 0.999 for both the Generator and the Discriminator, fixed the latent space dimensionality to *M* = 100 and sampled the random latent vectors from a multivariate Gaussian distribution centered at the origin with standard deviation equal to 1 in all *M* dimensions. Independently, we trained a Classifier using the training set, used crossentropy as loss function and a softmax as the activation function in the last layer, the ADAM optimizer with hyperparameters *η* = 3 ⋅ 10^−5^, *β*_1_ = 0.5, *β*_2_ = 0.99. The accuracy of the classifier on the test set reached ≈98.9%. Using the training set, we then trained the Encoder in a VAE and used the trained Generator as the Decoder. We used as loss function the Kullback–Leibler divergence and the hinge loss function. We also added as a regularizer the Classifier’s loss function and the Lagrange multiplier, λ, as hyperparameter set to λ = 100. During the training of the Encoder, we kept both the Generator and the Classifier fixed. In Fig 2 we show the training results. To train the NNs we used Flux [23] in Julia [24] and the code can be found in Ref. [25].

**Fig 3:**
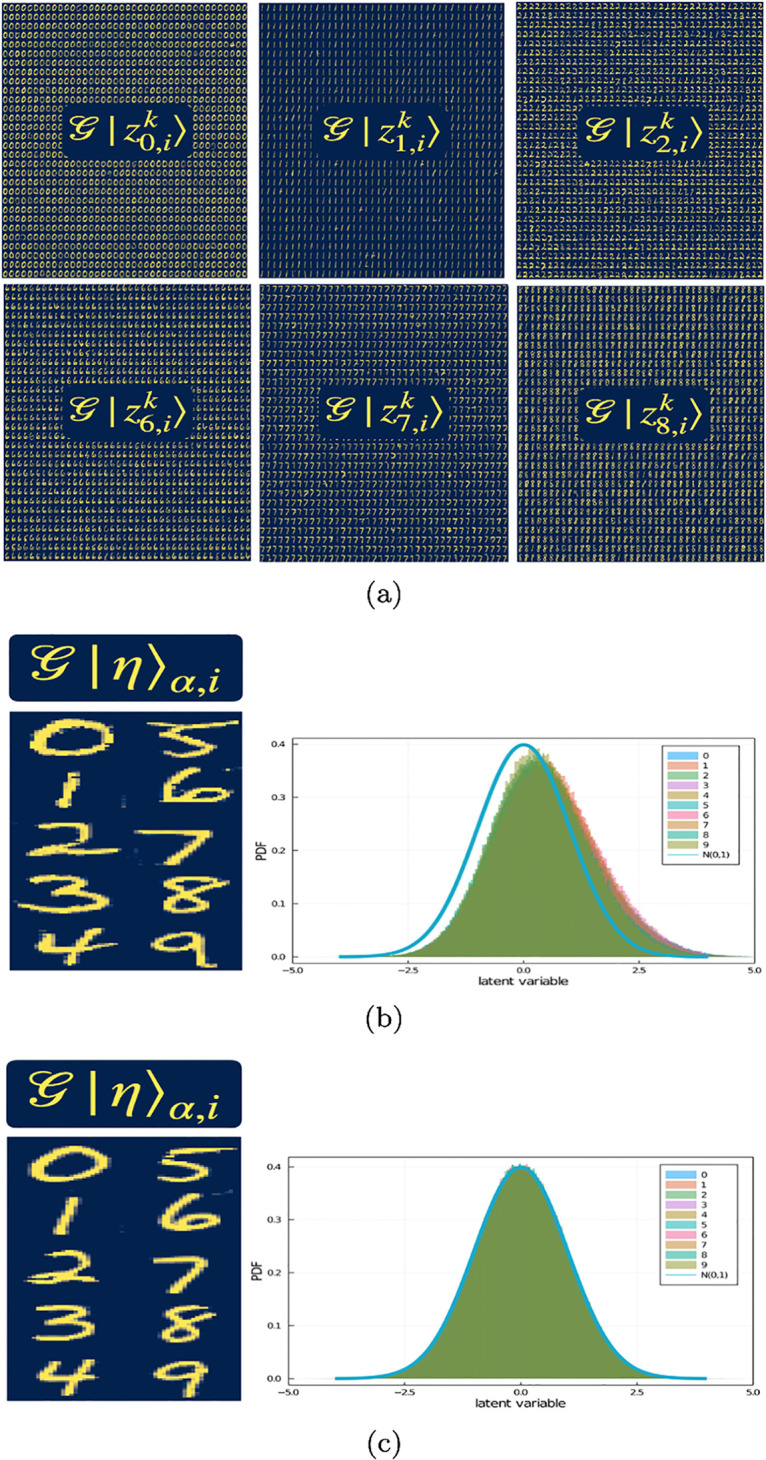
**a**) Samples of latent vectors $|z_{\alpha ,i}^{k}\rangle$, for labels *α* = 0, 1, 2, 6, 7 and 8. $G|z_{\alpha ,i}^{k}\rangle$ yields images of numbers with label *α*. We show 1200 latent vectors, per label, projected into real space. Average over the latent vectors per label yields |*η*〉_*α*,*i*_. **b) left panel** Decoded latent vectors |*η*〉_*α*,*i*_. The vectors |*η*〉_*α*,*i*_ were obtained as described in step 4(a). **b) right panel** The histogram for each label is Gaussian with non-zero mean. **c) left panel** Decoded latent vectors |*η*〉_*α*,*i*_. The vectors |*η*〉_*α*,*i*_ were obtained as described in step 4(b). **c) right panel** The histogram for each label is Gaussian with zero mean.

The latent space dimension is *M* = 100, while the number of labels is |*L*| = 10. Thus, following step 4, for each label we generated *n* = *M*/|*L*| sets of latent vectors, each set containing *V* = 5000 latent vectors. In Fig 3a we show a sample of latent vectors for labels 0, 1, 2, 6, 7 and 8, projected to real space using the Generator G. Then we take the average over each set as in step 5. We checked that the average and standard deviation over each of the entries in the set of vectors {|*η*〉_*α*,*i*_}_*α*,*i*_ converges. Interestingly, when taking the average over the set of latent vectors corresponding to a label and projecting back to real space, the label holds. For instance, in Fig 3b we show the projected image of the average over *V* for each set of latent vectors $\{|z_{\alpha ,i}\rangle {\}}_{k=1}^{V}$ in the case where the latent vectors were obtained following step 4(a), whereas Fig 3c corresponds to the case following step 4(b). We have also plotted the probability density function (*PDF*) per label in latent space for both cases and added a Gaussian distribution with mean and standard deviation equal to 0 and 1, respectively, for reference. Notice that the PDF in Fig 3b is shifted away from the Normal distribution, whereas in Fig 3c all PDFs are bounded by the Normal distribution, because latent vectors generated directly from latent space are, by definition, sampled from a multivariate Gaussian distribution with mean and standard deviation equal to 0 and 1, respectively. On the contrary, encoding real space vectors yields Gaussian vectors overall (*i.e*., the PDF over all latent vectors over all labels yields a Gaussian distribution, by definition) but the mean and standard deviation can differ from 0 and 1, respectively [4].

Step 4(a) gives robustness to this method and step 4(b) allows us to generate as many latent vectors as wanted with a specific label. Since the latent space dimension is *M* = 100, we need *M* averaged latent vectors |*η*〉_*α*,*i*_ to generate *M* orthogonal latent vectors. Since the number of labels is *α* = {0, …, |*L*| − 1}, then *n* = 10. To this end, we generate one set (*i.e*., *i* = 1) following step 4(a) and nine sets (*i.e*., *i* = 2, 3, …, *n*) following step 4(b).

**Fig 4:**
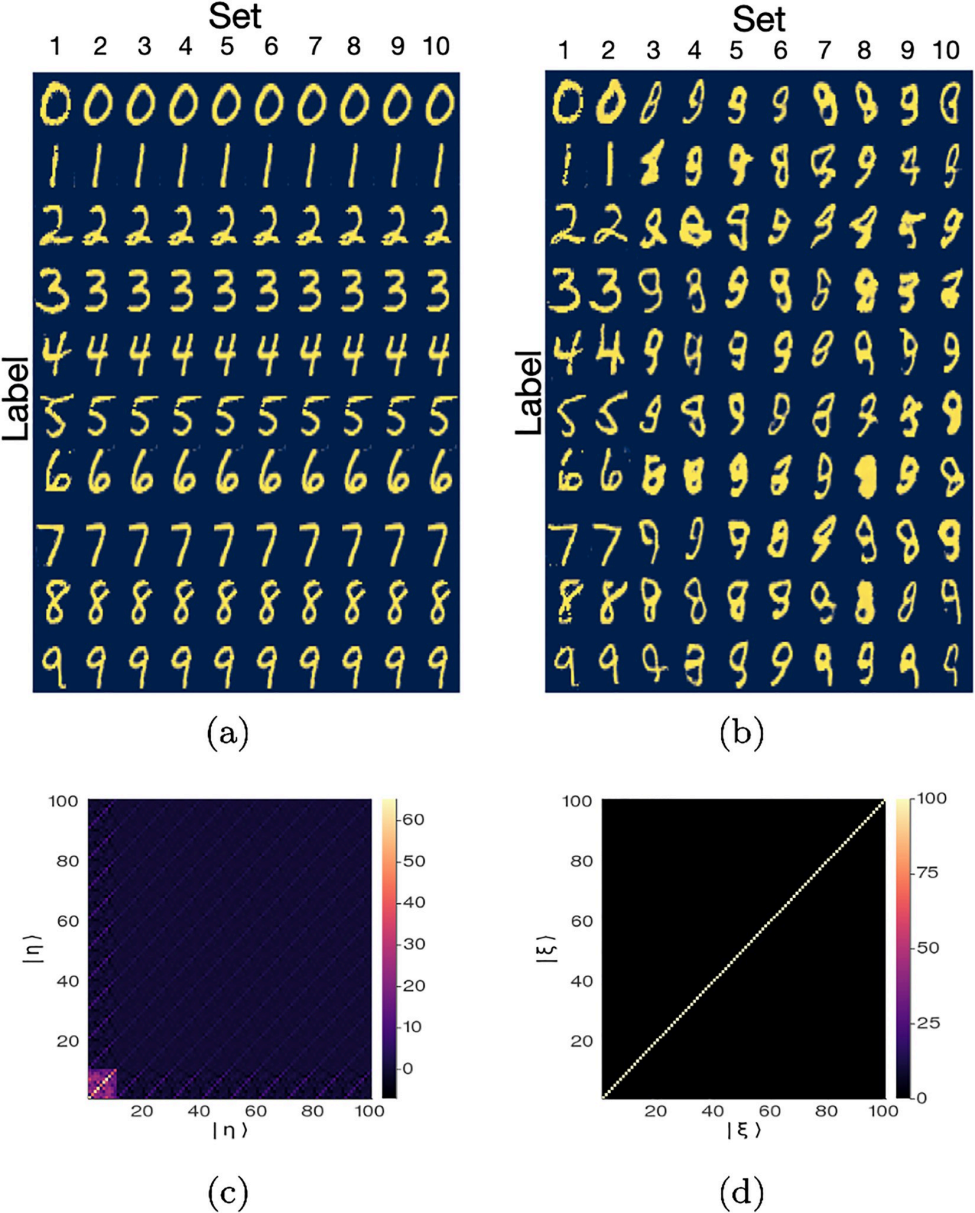
**a**) Projection to real space images of the latent vectors ${\{|\eta \rangle }_{\alpha ,i}{\}}_{\alpha =0,i=1}^{9,10}$ obtained as described in step 5. **b**) Projection to real space images of the quasi-eigenvectors ${\{|\xi \rangle }_{\alpha ,i}{\}}_{\alpha =0,i=1}^{9,10}$ obtained as described in step 6. The *α* index corresponds to the label (row) while the *i* index correspond to the set (column). **c**) The inner product of vectors ${\{|\eta \rangle }_{\alpha ,i}{\}}_{\alpha =0,i=1}^{9,10}$. **d**) The inner product of the quasi-eigenvectors ${\{|\xi \rangle }_{\alpha ,i}{\}}_{\alpha =0,i=1}^{9,10}$.

Fig 4a shows the projection to real space of all the |*η*〉_*α*,*i*_ vectors while Fig 4c shows the inner product _*α*,*i*_〈*η*|*η*〉_*α*′,*i*′_ as a heatmap, which shows they are non-orthogonal. At this point, we have *M* vectors |*η*〉_*α*,*i*_ in latent space each i) composed of the sum of *V* latent vectors, and ii) maps to a specific feature in real space (the image of a number). However, these vectors are not orthogonal. Using the Gram-Schmidt method described in step 6, we obtain a set of vectors, |*ξ*〉_*α*,*i*_, in latent space such that each |*ξ*〉_*α*,*i*_ vector i) encodes *V* latent vectors, ii) maps to a specific labeled feature (see Fig 4b) and iii) the |*ξ*〉_*α*,*i*_ vectors are orthogonal, as shown in Fig 4d). Since the Generator was trained using random vectors sampled from a multivariate Gaussian distribution centered at zero with standard deviation 1, the value of the norm of any random latent vector will be 〈*z*|*z*〉 ≈ *M*. Therefore, we fixed the norm of the quasi-eigenvectors to *C* = *M* (see Eq (8)).

Notice that while the non-orthogonal vectors |*η*〉_*α*,*i*_ for the MNIST GAN map to sharp images of easily-identifiable numbers in real space, not all quasi-eigenvectors map to images of numbers in real space. Only a few of the *M* linearly-independent directions in latent space (≈ 20) project to images of numbers in real space. We will show how to apply this property of the quasi-eigenvectors to the MNIST test set to classify images in latent space and to denoise real-space images. We also show how to build a rotation operator in latent space that generates feature transformations in real space.

## Results

### Using LSD as a classifier in latent space

We can express any latent vector |*z*〉, in terms of the quasi-eigenvectors, *viz*.

$$\begin{array}{c} \left|z\right\rangle =\sum_{k=1}^{M}c_{k}\left|\xi_{k}\right\rangle \;, \end{array}$$ (9)

where the coefficients *c*_*k*_ are given by,

$$\begin{array}{c} c_{k}=\left\langle \xi_{k}|z\right\rangle /C\;. \end{array}$$ (10)

**Fig 5:**
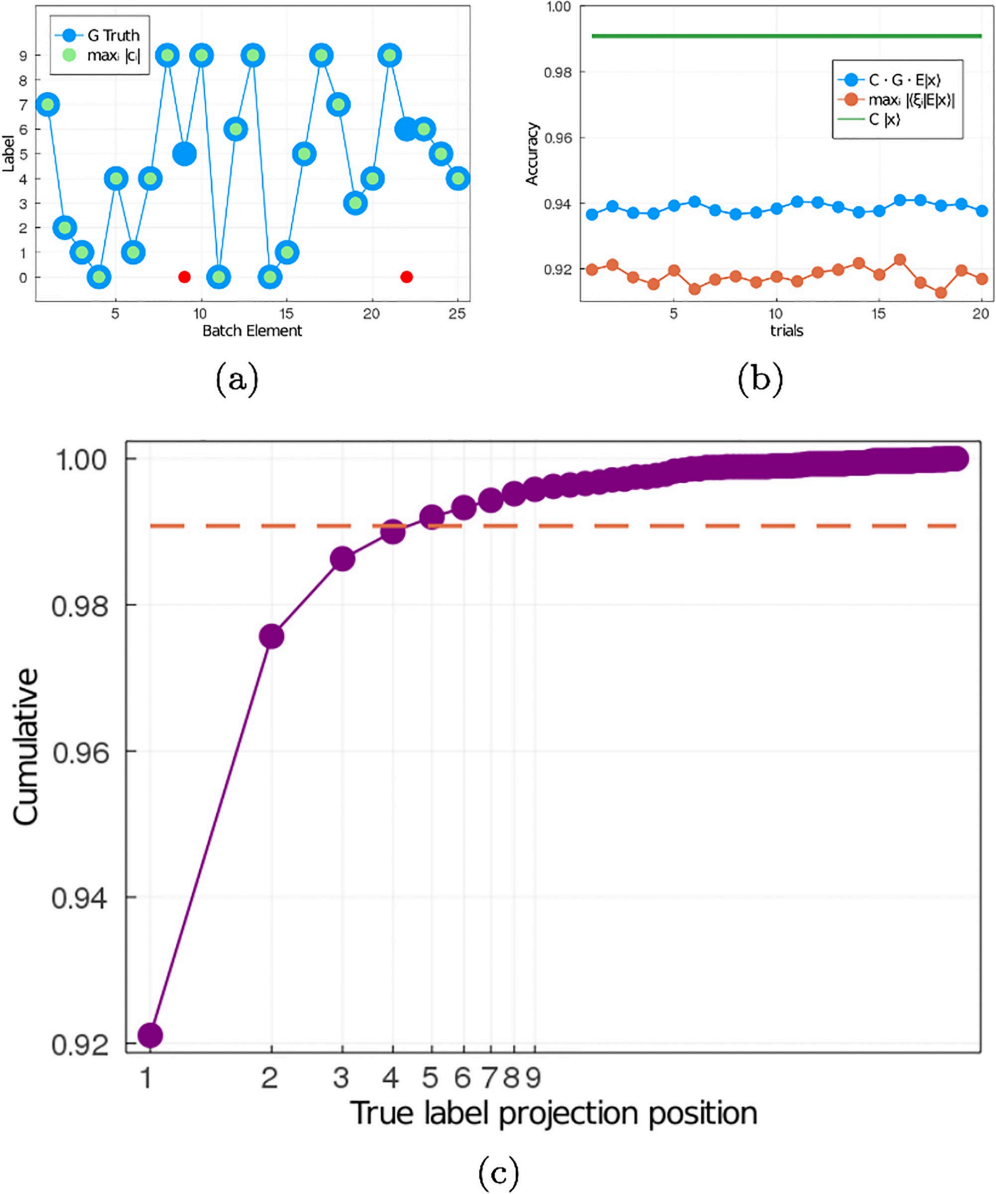
**a**) A batch of the MNIST test set classified by LSD using the largest amplitude. The largest amplitude |*c*_*i*_| corresponds to the quasi-eigenvector |*ξ*_*i*_〉 that contributes the most to the latent vector |*z*〉, and a subset of the quasi-eigenvectors map to each label one-on-one. Y axis corresponds to the label, X axis to the image in the batch. Blue dots, ground truth. Green (red) dots correspond to the case(s) where the label associated with the quasi-eigenvector with the highest amplitude is the correct (incorrect) label. **b**) Accuracy for different trials using the MNIST test set. The green curve is the Classifier’s accuracy (98.9%), the blue dots are the accuracy over the encoded-decoded MNIST test set (≈ 94%) and the red dots corresponds to the accuracy using the the largest amplitude in LSD (≈ 92%). **c**) Cumulative probability of the ground truth label being any of the *n* first largest amplitudes (X axis). For *n* = 1 the probability is 92%. The probability of the ground truth label being one of the labels with the 4 largest amplitudes is ≈ 98.9%, which is the classifiers accuracy.

Similar to principal component analysis, we are interested in how much information about an image is encoded in the quasi-eigenvector with the largest amplitude |*c*_*i*_|. We encode images from the MNIST test set into latent space, then express the latent vectors in terms of the quasi-eigenvectors (we call this expression *latent spectral decomposition* or *LSD*) and find the maximum amplitude |*c*_*i*_| for each latent vector. Recall that the amplitude |*c*_*i*_| is a measure of the projection of the latent vector with respect to the quasi-eigenvector |*ξ*_*i*_〉. Thus, the largest amplitude corresponds to the quasi-eigenvector that contributes the most to the latent vector. Since the quasi-eigenvectors are associated with labeled features in real space, we use the largest amplitude as a way to classify the image. Fig 5a shows a sample batch of 25 images. The blue dots corresponds to the true labels (see y axis), while the green (red) dots correspond to the case where label associated with the quasi-eigenvector with the largest amplitude is the correct (incorrect) label. In this batch, only batch elements 9 and 22 have true labels that do not agree with the label of the quasi-eigenvalue of the image with the largest amplitude. Since each time the Encoder encodes an image it generates a new random latent vector, then we could obtain a different outcome for batch elements 9 and 22 as well as the rest of the batch elements for each trial. For this reason, we perform an ensemble average over 20 trials. For each trial we take the whole MNIST test set and compute the accuracy of the latent space decomposition (LSD) classifier (see red dots in Fig 5b). We also computed the accuracy when the test set is encoded through the Encoder, then decoded through the Generator and finally classified (see blue dots in Fig 5b). We have included the accuracy of the trained Classifier in Fig 5b as an upper bound. While the trained Classifier has an accuracy of 98.8%, the LSD classifier has an average accuracy of ∼92%. This difference in accuracy, however, should not be interpreted as showing that the latent-space classifier does a poor job, but that the dominant few quasi-eigenvectors carry most of the information in latent space regarding the individual test-set images. In fact, the encoded 99% of the test-set data requires only the 10 linearly-independent directions in set 1, i.e., the largest amplitude correspond to quasi-eigenvectors in the first set.

**Fig 6:**
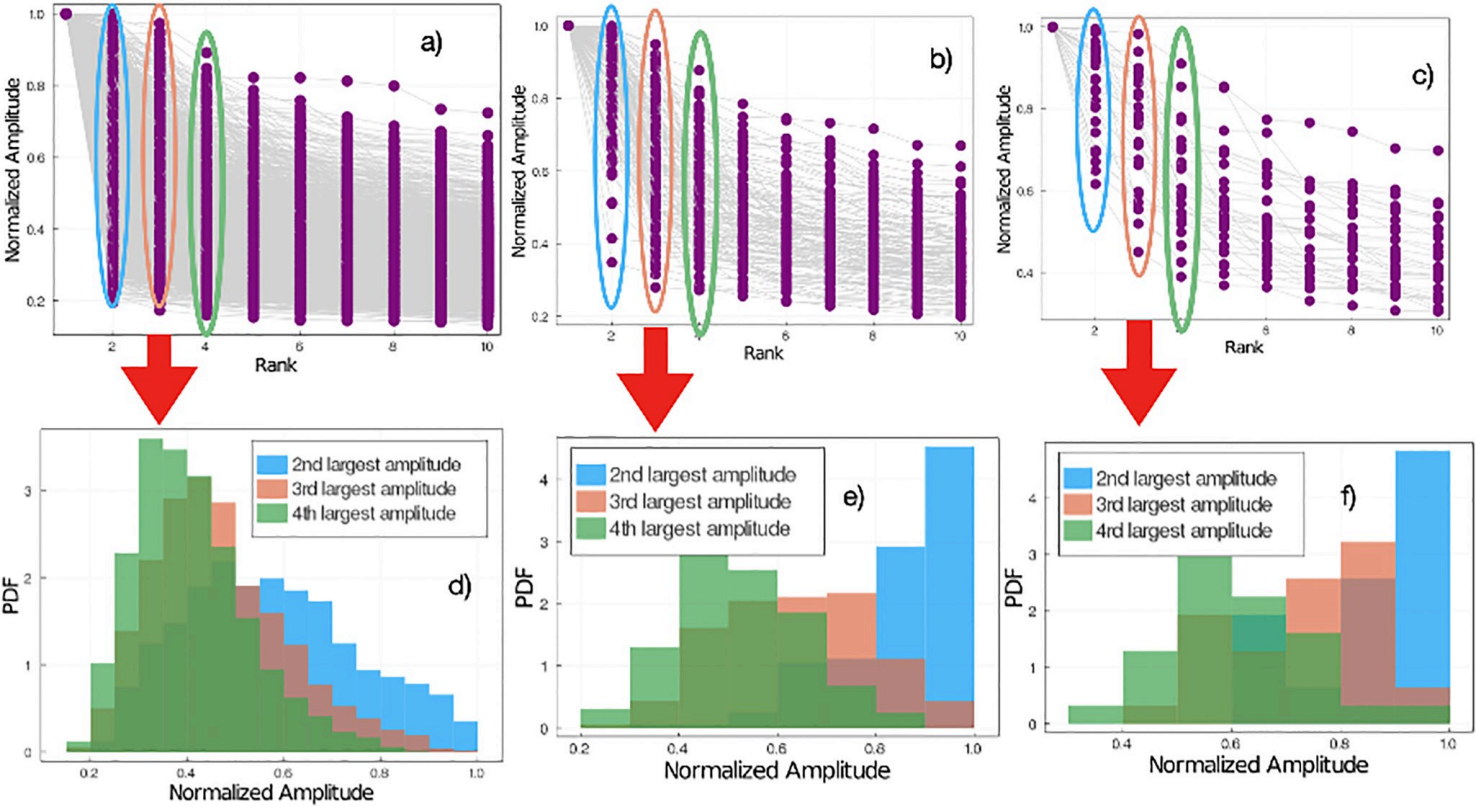
LSD normalized amplitude ranking for the cases where the true label corresponds to the largest amplitude quasi-eigenvector **a**), second largest amplitude **b**) and third largest amplitude **c**). Probability-density function of the second-, third- and fourth-largest LSD normalized amplitude when the true label corresponds to the largest amplitude **d**), second-largest amplitude **e**) and third-largest amplitude **f**).

Suppose that when we perform the LSD, we sort the amplitudes such that |*c*_1_| > |*c*_2_| > … > |*c*_*M*_| and ask *the position of the ground-truth label?* As previously mentioned, in 92% of the cases the ground-truth label corresponds to the first position (*i.e*., |*c*_1_|). In 5% of the cases the ground truth label corresponds to the second largest amplitude (*i.e*., |*c*_2_|). In Fig 5c we have plotted the cumulative of the probability for the ground-truth label being any of the first *n* positions. The dashed red line corresponds to the trained Classifier accuracy. Notice that the probability of the label being in position 1, 2, 3 or 4 of the LSD equals the accuracy of the trained classifier, *i.e*., in 98.9% of the MNIST test-set images the ground truth label is associated to a quasi-eigenvector such that the associated coefficient is either *c*_1_, *c*_2_, *c*_3_ or *c*_4_. In this sense, it is possible that even when the amplitude of the quasi-eigenvector associated to the ground-truth label is not the largest one, rather the 2nd or 3rd largest one, then |*c*_1_| ≳ |*c*_2_| or |*c*_1_| ≳ |*c*_2_| ≳ |*c*_3_|. To test this idea, in Fig 6 we have plotted the normalized amplitude (*i.e*., |*c*_*i*_|/max{|*c*_*j*_|}) *vs* the rank (*i.e*., sorted amplitudes from largest to smallest) for all images in the test set. Fig 6a corresponds to the images where the LSD amplitude of the quasi-eigenvector associated with the ground-truth label is the largest, whereas in Fig 6b and 6c the amplitude is the 2nd largest or 3rd largest, respectively. Given the large dataset, in Fig 6d–6f we have plotted the PDFs of the 2nd, 3rd, and 4th largest amplitudes for each of plots Fig 6a–6c. To be clear, from Fig 6a–6c we generated PDFs for the second-, third- and fourth-largest amplitudes in each plot and show the PDFs in Fig 6d–6f, respectively. Notice that when the largest amplitude corresponds to the ground-truth label (Fig 6a), the second-, third- and fourth-largest amplitude PDFs are centered below 0.6 (Fig 6d). When the second-largest amplitude corresponds to the ground-truth label (Fig 6b) the PDF of the second-largest amplitude is shifted towards 1, while the PDFs of the third- and fourth-largest amplitude amplitudes are centered below 0.7 (Fig 6e). Finally, in the case where the third-largest amplitude corresponds to the ground-truth label (Fig 6c), the PDFs of the second- and third-largest amplitude are shifted towards 1, while the PDF of the fourth-largest amplitude is centered below 0.7 (Fig 6f).

The previous results give us a broad picture of latent space topology: the labeled features project to well-defined compact domains in latent space. Let us now consider how we can use this information to denoise images.

### Denoising with LSD

The main issue when reducing noise in images is distinguishing noise from information. In this sense, a reliable denoiser has to learn what is noise and what isn’t. One reason deep generative models are promising for denoising data is that in optimum conditions the GM has learned the exact data distribution. Of course, if the data set has noise, the GM will also learn the embedded noise in the data set. However, by sampling the latent space we may find regions where the signal to noise ratio is sufficiently large. For large *M*, this sampling is computationally expensive. To avoid this cost, we propose to LSD as a denoiser.

**Fig 7:**
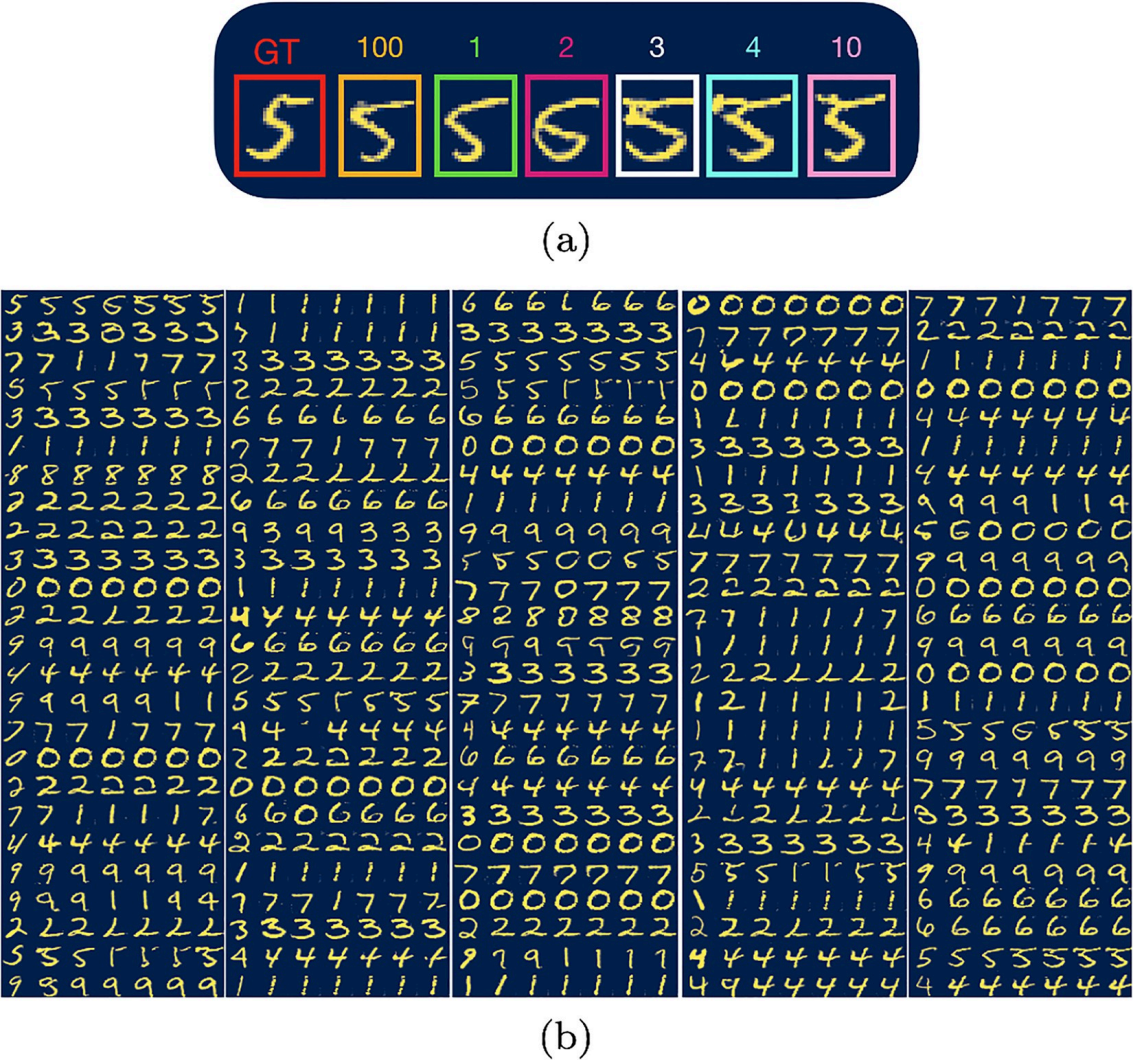
**a**) Image of the number 5 taken from the MNIST test set. The first image correspond to the ground truth (*GT*), the second image corresponds to the projected image of the 100 LSD components, the third, fourth, fifth, sixth and seventh images correspond to the projected images from the sum of the one, two, three, four and ten LSD components, respectively. **b**) 125 samples from the MNIST test set. Each sample is a row with seven images as shown in **a**).

Recall that in the previous section we showed that with a 98% accuracy the information needed to assign a label to the image is stored in either the first-, second-, third- or fourth-largest amplitude of the LSD. Therefore, we propose that once the test set is encoded into latent space, we decompose the latent vector in terms of the quasi-eigenvectors and drop the contribution from quasi-eigenvectors with low amplitudes. In Fig 7 we show the results of this truncation for 125 random sample images. In Fig 7a we describe how to understand these images. Fig 7b shows 5 columns, where each column has 25 rows and each row has 7 images. In each row, the first image corresponds to the ground-truth image, the second image is the image decoded from all 100 LSD components of the ground truth image. The third, fourth, fifth, sixth and seventh images are the images decoded after truncating the expansion after 1,2,3,4 and 10 LSD components of the ground truth image. In this method, denoising maintains the identity of the labeled feature in the image, *e.g*., each row shows different representations of the same number. In most cases in Fig 7, the denoised image looks clearer and sharper. However, sometimes the LSD components project back to the wrong number. However we can consider as many LSD components as the dimension of the latent space, so even if taking the first *n* LSD components yields the wrong number, taking the first *n* + 1 LSD components could yield the correct number. In the previous section we showed that using only the first 4 LSD components gave us a 98.9% chance of obtaining the right number.

### Operations in latent space

Here we explore how to build operators in latent space that can yield feature transformations in real space. Having a set of orthogonal vectors that span latent space allows us to perform most operations in latent space as a series of rotations, since we can express the operator as a superposition of the outer product of the quasi-eigenvectors. If we construct a rotation matrix, R, in latent space, we can then recursively apply R to a set of encoded images. After each iteration we project the output to real space to see the effect of the latent-space rotation. We can define a projection operator $B_{\xi_{i},\xi_{j}}$, such that,

$$\begin{array}{c} B_{\xi_{i},\xi_{j}}=\frac{1}{\left\langle \xi_{i}|\xi_{i}\right\rangle }|\xi_{j}\rangle \langle \xi_{i}|\;. \end{array}$$ (11)

This operator projects from |*ξ*_*i*_〉 to |*ξ*_*j*_〉, i.e., $B_{\xi_{i},\xi_{j}}|\xi_{k}\rangle =\delta_{\xi_{k},\xi_{k}}|\xi_{j}\rangle$, where $\delta_{\xi_{k},\xi_{k}}$ denotes the Kroenecker delta function. Similarly, we define the operator $R_{\xi_{i},\xi_{j}}\left(\Delta \theta ,\theta \right)$ as

$$\begin{array}{c} R_{\xi_{i},\xi_{j}}\left(\Delta \theta ,\theta \right)\propto (\text{cos}\left(\theta +\Delta \theta \right)|\xi_{i}\rangle +\text{sin}\left(\theta +\Delta \theta \right)|\xi_{j}\rangle )\\ \cdot (\langle \xi_{i}|\text{cos}\left(\theta \right)+\langle \xi_{j}|\text{sin}\left(\theta \right))\;, \end{array}$$ (12)

which projects from cos(*θ*)|*ξ*_*i*_〉 + sin(*θ*)|*ξ*_*j*_〉 to cos(*θ* + Δ*θ*)|*ξ*_*i*_〉 + sin(*θ* + Δ*θ*)|*ξ*_*j*_〉.

**Fig 8:**
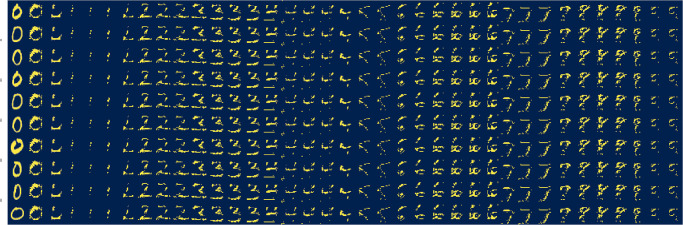
Ten latent vectors projected into real space after each iteration where the latent vectors are rotated an angle Δ*θ* ≈ *π*/6 from between two linearly independent directions associated with a quasi-eigenvector each. Rotations in latent space map to real space as label feature transformation, *i.e*., the images transform from the number 0 to number 1 and then from 1 to 2 until reaching number 9.

Starting from a set of images with label zero, we first encoded them to latent space, then we applied the rotation operator R recursively, as follows: First, we perform the rotation from the quasi-eigenvector associated with label zero to the quasi-eigenvector associated with label 1, *viz*., $R_{\xi_{\alpha =0,i=1},\xi_{\alpha =0,i=1}}\left(\Delta \theta ,\theta \right)$. Then, we performed a rotation from the quasi-eigenvector associated with label 1 to the quasi-eigenvector associated with label 2, *viz*., $R_{\xi_{\alpha =1,i=1},\xi_{\alpha =2,i=1}}\left(\Delta \theta ,\theta \right)$, and repeat *mutatis mutandi* until we reach the quasi-eigenvector associated with label *α* = 9. To keep the individual rotations in latent space small (and maintain the local linearity of the transforms), we fixed the rotation step size Δ*θ* ≈ *π*/6 so transforming from a direction associated with one quasi-eigenvector to a direction associated with a different quasi-eigenvector requires three sequential rotations. In Alg. 1 we show the pseudocode. To ensure the rotated latent vectors have constant norm value as in Eq (8), after each iteration we divide the latent vector |*z*〉 by $\sqrt{\frac{\langle z|z\rangle }{M}}$. After each iteration, we project the latent vector into real space. In Fig 8 we show this projection for a set of sample images. Notice how the numbers transform from 0 to 9. In principle, we could rotate through any other set of sequential features in this way. The key idea is that having a set of quasi-eigenvectors that span latent space each mapping to a specific label, we can define a metric in latent space defining the distance between the latent-space representation of each label.

**Algorithm 1**: Latent space rotation pseudocode.

initialization

|z〉=E|x〉 (initial condition)

Δ*θ* = *π*/3   (angular rotation step)

*α* = 0   (initial label)

*i* = 1   (set index)

**for** *α* *in* {0, 1, 2, …, 9} **do**

 **for** *r in* {1, 2, 3} **do**

$|z\rangle =R_{\xi_{\alpha ,i},\xi_{\alpha ,i}}\left(r\cdot \Delta \theta ,\left(r-1\right)\cdot \Delta \theta \right)|z\rangle$ (rotation)

$|z\rangle =|z\rangle /\sqrt{\frac{\langle z|z\rangle }{M}}$ (norm)

|x〉=G|z〉 (projection to real space)

 **end**

**end**

## Discussion

We have shown that it is possible to build a set of orthogonal vectors (quasi-eigenvectors) in latent space that both span latent space and map to specific labeled features. These orthogonal vectors reveal the latent space topology. We found that for MNIST, almost all the images in the data set map to a small subset of the dimensions available in latent space. We have shown that we can use these quasi-eigenvectors to reduce noise in data. We have also shown that we can perform matrix operations in latent space that map to feature transformations in real space.

On the one hand, the deeper the NN the better its capacity in learning complex data and as depth increases, the non-linearity increases as well. On the other hand, it has been proposed the equivalence between deep NNs and shallow wide NNs [16]. From catastrophe theory [26], we know that in non-linear dynamical systems small perturbations can be amplified leading to bifurcation points leading to completely different solution families of these non-linear dynamical systems. The results in Ref. [17] suggest a different picture with what the authors call *vector arithmetics* in which adding or subtracting vectors in latent space can yield a feature addition, removal or modification (*e.g*., hair color, sunglasses, facial hair in the case of a headshot image data set). This behavior hints at the possibility of building a vector basis in latent space. It is not obvious why or how the label embeddings cluster in latent space or why they do so in a linearly independent manner. To put it in different terms, it would appear that the training of the GAN is reminiscent of a symmetry breaking mechanism from a rotationally invariant latent space to one where the label embeddings are linearly independently clustered. We consider that understanding why this pattern of clustering occurs is of great relevance and we intend to explore it in future work. Our intuition behind using the Gram-Schmidt method comes from the latent-space vector arithmetic [17] and the flexibility of the method whereby one first chooses a set of vectors from which the vector basis is built.

Our work contributes to this discussion of the emergent effective linearity of NNs as transformations. While the NNs we used are intrinsically non-linear, they exhibit local linearity over a region of interest in latent space. This subspace maps to labeled features. In this sense, we say the non-linear NNs are effectively linear over the domain of interest. As a proof of concept, we have shown this for MNIST successfully, and our results serve as a proof of concept. Future work is aimed at testing this method in broader data sets, such as, CIFAR [27] and ImageNet [28]. Similarly, we plan to test this method for different latent space dimensionality and the effect it can have on feature entanglement.

We have considered labeled data which is a strong assumption in real problems since it is usually difficult to have that type of information. However, having a set of quasi-eigenvectors potentially allows us to recreate unlabelled data through latent superposition. We have not tested this here and we leave it for further work as well as testing this framework in other well-known datasets. Fundamentally, we have shown that the data clustered in the GAN’s latent space is linearly independent by building a set of quasi-eigenvectors pointing to each of these clusters. Further work is needed to understand the relationship between labels and linearly-independence when the latent space dimensionality varies. The classifier and encoder were merely tools used to be able to span latent space and further work is aimed at simplifying this framework.

From an application standpoint, mapping to dominant quasi-eigenvectors could be useful for medical imaging, diagnosis and prognosis if, *e.g*., the labels denoted the severity of a disease; for predicting new materials if the labels denoted specific material features or external physical parameters.
